# Glutamate and clock help bean bugs track seasonal reproductive changes

**DOI:** 10.1371/journal.pbio.3001796

**Published:** 2022-09-07

**Authors:** Sergio Hidalgo, Joanna C. Chiu

**Affiliations:** Department of Entomology and Nematology, College of Agricultural and Environmental Sciences, University of California, Davis, California, United States of America

## Abstract

Animals adapt their seasonal physiology by measuring photoperiodic changes over the calendar year, but the neuronal mechanisms conveying this information are unclear. This Primer explores a new PLOS Biology study that sheds light on this process by uncovering changes in glutamate dynamics that are dependent on photoperiod and a clock gene.

## Teaser

Animals adapt their seasonal physiology by measuring photoperiodic changes over the calendar year. A new study in *PLOS Biology* uncovers changes in glutamate dynamics in the bean bug that are dependent on photoperiod and a clock gene.

Organisms adapt to seasons to survive. This is a widespread phenomenon that relies predominantly on the ability to measure changes in daylength (i.e., photoperiodism). Studies in insects have helped to understand this process, as photoperiod is used by many insects to regulate adaptations such as migration and diapause [1]. The bean bug *Riptortus pedestris*, for instance, shows a clear photoperiodic response in reproduction. While it remains reproductive under long photoperiod, it enters robust diapause under short photoperiod. A long-standing hypothesis proposed by Erwin Bünning in 1936 suggests that the same molecular machinery used by organisms to tell time over the day–night cycle, the circadian clock, is required for photoperiodism [2]. Hormonal changes in specific brain areas, including the pars intercerebralis (PI), are required to terminate overwintering and promote development. Yet, the neuronal pathways used by the clock to convey photoperiodic cues to the PI and other brain regions involved in photoperiodism have not been fully elucidated [2,3].

A new study published in *PLOS Biology* by Hasebe and Shiga now shows that extracellular glutamate level is sensitive to photoperiod [4]. An increase in extracellular glutamate is observed in brains from bean bugs reared in short photoperiod compared to those reared in long photoperiod. This change is dependent on the clock gene *period* (*per*), consistent with previous studies showing the importance of *per* in photoperiodism [5,6]. These results identify a new pathway by which the circadian clock could relay photoperiodic information to regulate seasonal biology.

**Fig 1:**
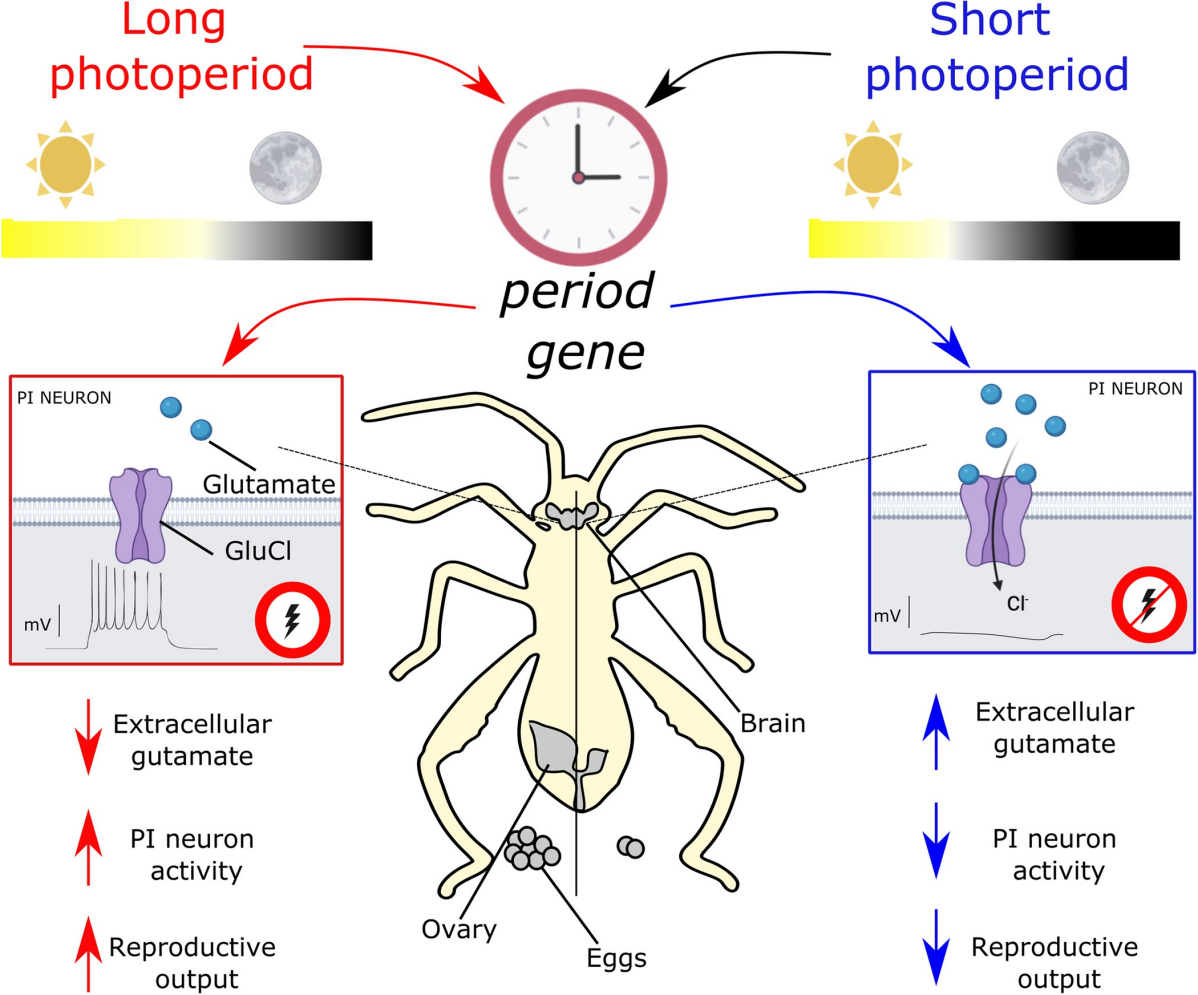
Circadian clock gene *period* regulates photoperiodic-dependent accumulation of glutamate to modulate seasonal physiology. Brain extracellular glutamate in bean bug is increased under short photoperiod, and this event is dependent on *period* expression. This increase inhibits the neurons in the pars intercerebralis (PI) through activation of the glutamate-dependent chloride channel GluCl. Glutamate dynamics underlie the photoperiod-dependent change in the firing activity of the PI neurons from extremely active in long photoperiod to silent in short photoperiod. Consequently, changes in PI activity regulate adaptations in seasonal reproductive outputs, in particular, egg laying.

The authors manipulated the expression of glutamate-metabolizing enzymes glutamate oxaloacetate transaminase (*got*) and glutamine synthetase (*gs*) to test the functional relevance of glutamate dynamics. Knocking down *got* or *gs* altered brain glutamate levels and impaired photoperiodic induced changes in reproduction. This translates into defective adaptation as the bugs are unable to enter full diapause while exposed to short photoperiod and to fully promote reproduction under long photoperiod. In a previous study, the authors showed that the electrical activity of bean bug PI neurons is responsive to photoperiod [6]. In long photoperiod, PI neurons display bursts of activity while they go silent in short photoperiod. Knockdown of either *got* or *gs* also prevents changes in firing patterns consistent with a role of glutamate in this process. Glutamate can act as an inhibitory signal in insects through the activation of the glutamate-gated chloride channel GluCl. Upon activation, GluCl triggers the influx of chloride ions, silencing the cells. Thus, the elevation of extracellular glutamate in short photoperiod could potentially explain the change in activities of PI neurons. Consistent with this notion, exogenous glutamate application inhibits PI neurons activity and knockdown of *got* or *gs* ablates the photoperiod-dependent difference in firing. The authors suggest an involvement of GluCl in photoperiodic response as they show that the inhibition triggered by glutamate in PI neurons is dependent on chloride ion conductance. This was further tested by knocking down *glucl* and observing blunted photoperiodic responses at the levels of PI firing pattern and reduced reproductive diapause. Taken together, their results support a direct role of glutamate in PI neurons to mediate photoperiodic responses (Fig 1).

The findings from Hasebe and Shiga are exciting, as they propose a mechanism in which the circadian clock influences the PI and potentially triggering hormonal changes required for seasonal adaptations. Interesting new questions arise from this study. For instance, what is the molecular mechanism triggering the increase in extracellular glutamate in short photoperiod? First, it is possible that overall glutamate level is elevated, thus increasing its extracellular levels due to increased production or reduced metabolization of the neurotransmitter. This appears to be unlikely as neither *got* nor *gs* expression changes with photoperiod [4]. This does not rule out, however, that the activity of the enzymes could change. Second, it is possible that the ability of the system to clear glutamate is reduced. Upon release in the synaptic cleft, neurons and glia cells can reuptake glutamate by the excitatory amino acid transporters (EAATs), expressed in the cell membrane. A reduction in EAATs expression, in either neurons or glia, could therefore modulate extracellular glutamate levels. Finally, and close to the authors proposed explanation, vesicular glutamate transporter (VGlut) could be involved. VGlut functions in both neurons and glia, loading glutamate into synaptic vesicles. An increase in VGlut levels during short photoperiod would potentially trigger an increase in extracellular glutamate by increasing the glutamate amount in the vesicles. Hasebe and Shiga provide exciting data showing an increase of *vglut* expression in short photoperiod, in line with a previous study showing that diapause in the bean bug relies on *vglut* expression [7]. Further experiments are needed to explore these possibilities.

It is also unclear whether glutamate dynamics arise from a widespread event or whether they correspond to changes in only a few neurons connecting to the PI. Previous studies in other insects have shown an important role of the *per*-expressing small ventral-lateral neurons (s-LNvs) and the dorsal neurons-1 (DN1) in photoperiodism [8,9]. These neurons form part of the circadian clock neuronal network and interact with each other modulating their activity through the neuropeptide Pigment Dispersing Factor (PDF) and glutamate [10]. Interestingly, it has been shown that DN1 neurons express *vglut* and are functionally connected to the PI neurons in *Drosophila*, making this cluster a good candidate for the *per-*dependent effect [10].

Finally, the role of the clock gene *per* in glutamate dynamics could be mediated by *per* function in the circadian clock (i.e., modular pleiotropy) or by its function as an independent element (i.e., gene pleiotropy) [1,3]. Studies assessing this distinction have been equivocal. Consistent with a modular pleiotropy, knocking down *cycle*, another core clock component, or *per* affect both seasonal diapause and circadian cuticle deposition in the bean bug [5]. In contrast, studies in *Drosophila melanogaster* have shown that *per* mutants are still able to sense photoperiods [1]. It would be interesting to explore if glutamate dynamics are present in other species and can be similarly ablated by knocking down clock components to further dissect the role of the circadian clock in photoperiodism.

## Abbreviations

DN1: dorsal neurons-1
EAAT: excitatory amino acid transporter
got: glutamate oxaloacetate transaminase
gs: glutamine synthetase
PDF: Pigment Dispersing Factor
per: *period*
PI: pars intercerebralis
s-LNvs: small ventral-lateral neurons
VGlut: vesicular glutamate transporter
